# Sexual Harassment in Fitness Instructors: Prevalence, Perpetrators, and Mental Health Correlates

**DOI:** 10.3389/fpsyt.2021.735015

**Published:** 2021-10-29

**Authors:** Therese Fostervold Mathisen, Nina Sølvberg, Christine Sundgot-Borgen, Jorunn Sundgot-Borgen

**Affiliations:** ^1^Department of Health and Welfare, Østfold University College, Fredrikstad, Norway; ^2^Institute of Sports Medicine, Norwegian School of Sport Sciences, Oslo, Norway; ^3^Regional Department for Eating Disorders, Division of Mental Health and Addiction, Oslo University Hospital, Oslo, Norway

**Keywords:** mental health symptoms, depression, anxiety, eating disorders, personal trainer, group instructor, sexual harassment

## Abstract

**Introduction:** This paper explores frequency of experiences of sexual harassment (SH) among fitness instructors, outlines the typical perpetrator, and explores associated mental health symptoms.

**Design:** Cross-sectional survey.

**Materials and Methods:** A total of 270 Norwegian fitness instructors, recruited in social media, responded to an electronic questionnaire in 2019/2020 on experiences of body appearance pressure, SH, and mental health symptoms. Outcomes included in this publication are experiences of SH, and symptoms of mental health issues based on the Hopkins symptom checklist, SCL-10; Beck Depression Inventory, BDI-1a; and Eating Disorder Examination Questionnaire, EDE-q.

**Results:** The frequencies of SH experiences were 30% among 211 women and 22% among 59 men, respectively, with customers being the most frequent source of such approaches and personal trainers (PTs) more often reporting such experiences compared to group instructors (GIs) and to those operating as both PT and GI. Women having experienced SH had significantly higher scores in symptoms of depression, anxiety, and eating disorders compared to women with no such experience.

**Conclusion:** The high frequency of SH experiences among fitness instructors, with customers emerging as perpetrators and typically in the context of personal training, necessitates increased awareness of SH in the fitness industry. To reduce the occurrence of SH, the fitness centers need to communicate countermeasures with high level of compliance.

## Introduction

Sexual harassment (SH) concerns a subjective perception of unwanted sexual attention (1). It is a multifaceted concept and may be classified in three different categories: verbal, nonverbal, and physical SH (2). Verbal SH includes unwanted sexual comments or remarks about your body, appearance, or private life; messages with sexual content; or suggestions for sexual services, in either oral, written, or digital nature. Examples of nonverbal SH are being exposed to unwanted sexual glances, body movements, indecent exposure, persecution, or spreading of private or intimate pictures. Physical SH includes involuntary hugging, kissing, or other types of unwanted physical toughing, including rape or attempted rape (2, 3).

A common understanding has been that SH occurs in relationships where the perpetrator has a higher position on the hierarchical ladder (4). The implication from this may be that workplaces are settings in which SH may easily occur (i.e., the organizational theory) (4). A national survey on living conditions at work in Norway reveals that 4% experience SH regularly (i.e., monthly or more often), with women (7%) more exposed than men (2%) (results from responding to question “Does it happen that you are exposed to unwanted sexual attention, comments or the like at your workplace?,” rating their response from “yes, weekly,” yes, monthly,” or “no”) (5). Nuanced analyses further point to a highest frequency of SH among workers within health and restaurant services (~24% of women and ~21% of men) (5). The difference in experiences of SH between sexes and the higher frequency within the health services are also emphasized in other studies (2, 6, 7). While an explanation to the high frequency of SH in health services may be the close and often intimate interaction with clients, the higher frequency of SH in women may be due to the fact that there is a higher number of women working within health services (8). Nevertheless, meaningful prevalence rates of workplace SH are lacking, mainly due to methodological differences and limitations related to the septicity of samples examined (3). Most research on workplace SH has examined intra-organizational harassment, i.e., harassment from colleagues, leaders, or others working within the organization (1). SH perpetrated from persons outside the organization is less studied, but recent research shows that customers, clients, and patients are frequently the perpetrators, especially within service-oriented and healthcare professions (2, 6, 7). This can be understood as a continuation of the higher hierarchical position by the perpetrator, as customers and clients are paying for services offered. In such a context, the perpetrator may perceive the consequences (e.g., being accused, being prosecuted, being dismissed from the workplace) from acting small and may easily get away with no further means, or simply only facing an official complaint, specifically if there is low organizational priority of measures against such behavior (4, 9, 10).

Similar explanations may apply to between-colleague harassment, where SH may be motivated by body objectivization within a milieu (11) and where there is no or low risk of penalties. As such, a context of increased risk for exposure to SH is portrayed, explained by the four-factor theory, being a “multi factor theory” resting on four explanatory elements. First, the perpetrator is motivated for SH (e.g., driven by power/control and/or sexual attraction); secondly, experiences of internal inhibitions are low (overcomes any moral restrains); third, there are a lack of external inhibitions (this relates to organizational workplace barriers, like clear communication on measures toward SH and/or emphasizing gender equality); and finally, the victim reflects low assertiveness or organizational position (12).

For decades, the marketing and promotion of fitness centers, and the services within the centers in particular, has been centered on objectivization of the body (13, 14). Specifically, for women this may have contributed to underline the sex role that society traditionally has prescribed for women (i.e., as a sexual object), which may facilitate lower threshold toward sexually related verbal, behavioral, or physical acts (12). Furthermore, fitness centers are industries where physical appearance and bodily movement are naturally in focus and often verbally or nonverbally reacted upon. As such, unwanted sexual attention or behavior may easily occur, covered by a culture in which the conduct may be judged normal, and within an organization in which countermeasures seems to be less prioritized, or at least not strongly communicated (12). Nevertheless, there is scarce knowledge on the extent of SH in the fitness industry. We recently reported that a high frequency of fitness instructors experienced body appearance pressure (15), which points to a serious consequence of working within an industry where body appearance has been idealized for decades (13, 16). Working on profit-based contracts, relying on a “bodily capital” (i.e., relying on body appearance to win customers, as this may promote credibility on health and exercise knowledge) (14), may increase the risk for experiences of SH. Further, being dressed in tight clothes (increasing the risk of body objectivization) (17) and instructing detailed exercise techniques demanding physically close interactions, similar to the close client interactions typical for those working in other health services (increasing the likelihood of unwanted physical contact) (4), seem reasonable to suggest that fitness instructors may be at specifically high risk of SH experiences. As an extension to this, it may be hypothesized that offering personal exercise instruction (e.g., personal training) may increase the risk to experience SH compared to instructing groups of participants.

Negative consequences of experienced SH at work may vary depending on the type of harassment, who the perpetrator(s) was, the personal traits of the individual who experienced SH, where it happened, and how frequently it happened. It may be personal consequences causing serious mental strain, and financial difficulties or work-related consequences like job loss (2, 3, 10). Concurrently, findings suggest that specifically working in physical professions and in personal service professions (“blue collar workers”), the professionals tend to be less frequently affected by SH (despite more frequent experiences of SH compared to “white collar workers”) (9). As such, they seem to accept the incidents as part of their work and/or ignore it (9), which is underlined by findings suggesting a diversity of responses in coping with, and tackling, experiences of SH, but uncertainty as to what circumstances the different strategies apply (4). Hence, in an industry that for so long has objectified the body, with many recruited instructors often continuing manifesting this objectivization through visual communication in social media or by promoting services focused on shaping a perfect body figure (14, 18), it is difficult to say how experiences of body objectivization (here, behavior that may be experienced as SH) affect the individual.

Little is known on the prevalence of, and associations to, mental health from experiences of SH among fitness employees. The aim of this paper is to explore the frequency of experiences of SH at the work arena among female and male fitness instructors, and to study any differences between sex, and between personal trainers (PTs) and group instructors (GIs), and to study the origin of the perpetrator of SH in the fitness center arena. Additionally, this paper aims to explore any association between experiences of SH with mental health symptoms including symptoms of eating disorders. Considering the previous findings of high frequency of SH in health service professions, women more typically being offended compared to men, clients compromising a frequent perpetrator in health services, and resting on the four-factor theory (4), we hypothesized the following: (1) the frequency of SH in fitness instructors is high and similar to previous findings from health and service workers, (2) women more frequently experience SH than men, (3) PTs experience more SH compared to GIs, (4) clients are the most typical perpetrator, and (5) experiences of SH associate with more severe symptoms of psychopathology (here, symptoms of depression, anxiety, and eating disorders).

## Materials and Methods

### Design

We recruited for a cross-sectional study of PTs and group fitness instructors in Norway between November 2019 and March 2020. The study aimed to explore body figure idealization, experiences of body appearance pressure, and SH in fitness instructors of different sex and professions. For the purpose of this paper, only results related to SH are presented. All participants responded to an electronic questionnaire, estimated to take 40 min to complete with opportunities to pause, and having the topics within the questionnaire clearly described in an informed consent. There was no compensation offered for participation.

### Procedure

CEOs or the heads of instructors in fitness centers in Norway were asked to distribute recruitment information by email and poster to the fitness instructors, and concurrently we also recruited participants through interest groups for instructors in social media (Facebook).

**Figure 1:**
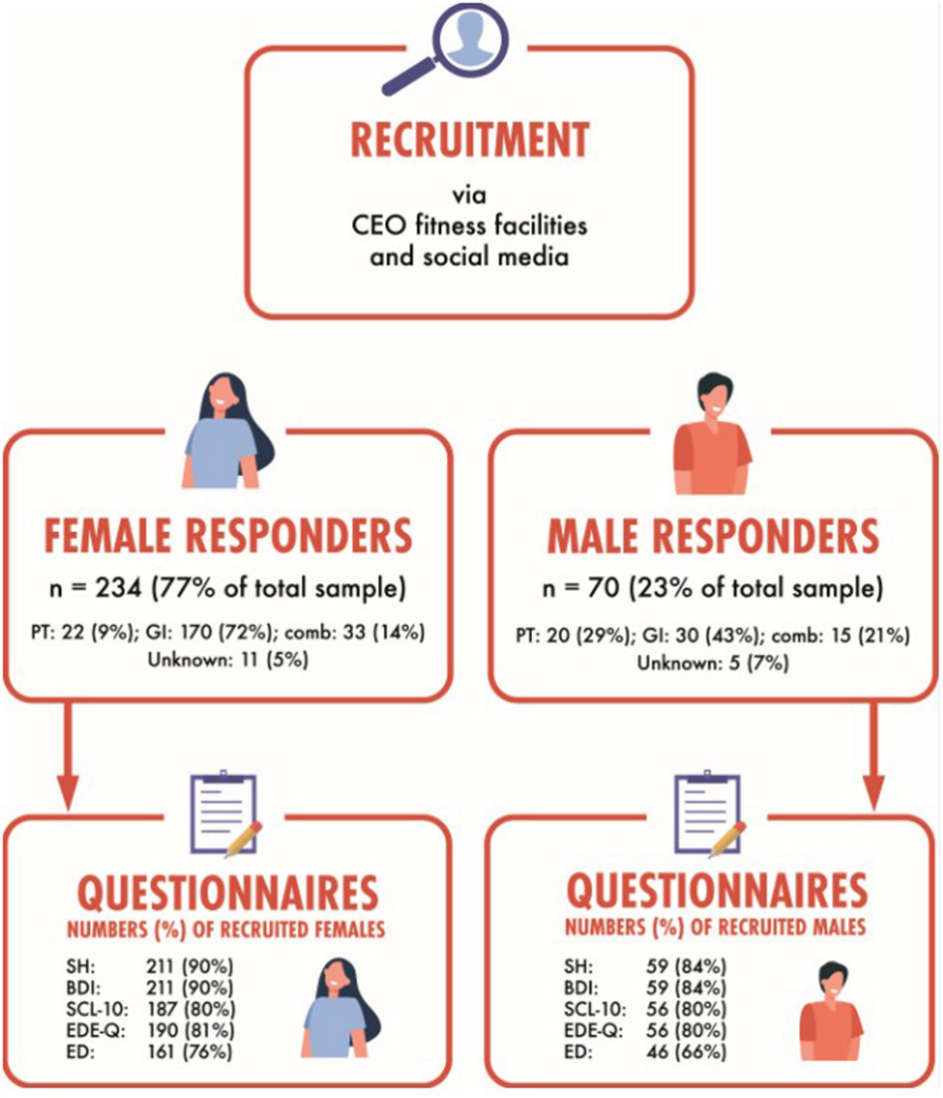
Number of participants recruited and responding for the different questionnaires, separated by sex. CEO, chief executive officer; PT, personal trainer; GI, group instructor; comb, combined profession of PT and GI; SH, sexual harassment questionnaire; BDI, Beck Depression Inventory; SCL-10, Hopkins Symptom Checklist 10 items; EDE-q, Eating Disorder Examination Questionnaire; ED, self-reported history of eating disorders.

We invited fitness instructors to participate in a survey on motives and attitudes toward physical activity, diet, and body appearance pressure, and experiences of SH. To qualify for participation, respondents had to operate as a PT or GI, or a combination of both (combo) in any fitness center facility at the time of recruitment, and to understand Norwegian written language. The numbers recruited and included are presented in Figure 1. Findings on experiences of body figure idealization, experiences of body appearance pressure, compulsive exercise, and prevalence of symptoms on mental health issues have been published elsewhere (19, 20). Results on SH are presented herein, with median scores on symptoms of depression, anxiety, and eating disorders provided for secondary analyses.

### Questionnaires

The first part of the survey asked for demographic information of which the following is presented in the current paper: age, education, profession, and BMI. To keep the comprehensive questionnaire short, we chose to use shortened, validated versions of questionnaires on mental health when available.

#### Sexual Harassment

Experiences of SH were explored by asking for any experience of such in current work position by using three categories (i.e., verbal, nonverbal, and physical SH), all with response rates of “no” or “yes,” with the latter differenced into “from customers,” “from colleagues,” and “from leaders.” In lack of a consensus or common definition of the concept “sexual harassment,” we applied previously suggested definition of SH and the categorization of such (2). First, SH was defined and explained as “any undesirable sexual attention which was experienced as offensive, frightening, hostile, degrading, humiliating or troublesome. The attention can take the form of unwanted sexual comments, glances, messages, spread of images, touches, indecent exposure of others, persecution, or physical abuse.” Next, experiences of SH were divided into the three categories: (1) have you experienced any unwanted sexual comments on your body, appearance, or private life, received other messages with sexual content, or received suggestions for sexual services (messages by oral, written, or digital nature)? (verbal); (2) have you been exposed to glances, sexual movements, indecent exposure of others, persecution, or spread of private, intimate pictures? (nonverbal); and (3) have you been experiencing involuntary hug, kisses, or other physical touches, or rape/attempted rape? (physical).

#### Beck Depression Inventory, Version 1a (BDI-1a)

The BDI-1a (Cronbach's α in the current study 0.88) measures current (past week) self-reported symptoms of depression (21). It consists of 21 items scored on a four-point Likert scale ranging from 0 (not at all) to 3 (extreme). The total score range is 0–63, and a cutoff score of ≥21 is recommended to detect a clinically significant episode of major depression (21).

#### Hopkins Symptom Check List (SCL-10)

The SCL-10 (Cronbach's α in the current study 0.90) is the short version of the SCL-25 (originally the short form of SCL-90), which validly and reliably measures symptoms of depression (six items) and anxiety (four items) (22). It consists of 10 questions scored from 0 to 4, and all are averaged into a global score, in which higher scores present higher severity of symptoms. A cutoff score of 1.85 indicates symptoms of mental health issues (22).

#### Eating Disorder Examination Questionnaire (EDE-Q)

The EDE-q (Cronbach's α in the current study 0.95) is a self-report questionnaire measuring the symptoms of eating disorders (23). It consists of 28 questions, of which 22 questions are scored on a Likert scale from 0 to 6 and divided into four subscales (shape, body weight, and eating concerns, and eating restriction). The subscales are averaged into a global score. Six questions measure the frequency of binge eating and purging behavior. An EDE-q global cutoff score of 2.5 has previously been found to identify the probability of having an eating disorder in a Norwegian sample (24).

### Ethics

This study was approved by the Norwegian Regional Committees for medical and health research ethics (No. 28855) and the Norwegian Centre for Research data (No. 868768) and was registered in the Clinical Trial registry (No. NCT04135729). The participants consented to participation by responding to an email containing study information, clearly describing the topics within the questionnaire, followed by signing a letter of informed consent. The consent and questionnaire were made in the web-based system SurveyXact 8.2 offered by Ramböll, Aarhus, Denmark. Considerations were made with regard to the risk–benefit for harms or regrets from participating in a questionnaire-based survey including sensitive information. This questionnaire-based survey was continued inasmuch as the rationale was explicitly prospectively explained to participants by written invitation, participation was anonymous and voluntary, and by having previous positive evaluations on risk–benefit ratios regarding sensitive data contribution in surveys (25).

### Statistics

All data were analyzed with SPSS version 26 (IBM, Armonk, NY, USA). Data were visually inspected for normality and presented as median (99% confidence interval, CI) as none were normally distributed, except for demographic data, which are presented as mean (Sd) and range and with a significance level of 0.05. We compared the frequency of experience of SH between women and men (*hypothesis 2*) in addition to comparisons between exercise profession (e.g., PT and GI) (*hypothesis 3*). We further compared whether customers, clients, or leaders were more frequently reported as perpetrators (*hypothesis 4*) and finally explored if there were any differences in symptoms of mental health issues between those who had experienced SH and those with no such experience (separately by sexes) (*hypothesis 5*). Categorical data were compared by Pearson's chi-squared test, while continuous data were compared by Mann–Whitney U. Considering the explorative approach of this study, a Bonferroni correction was considered too conservative; hence, *p* ≤ 0.01 were evaluated as statistically significant for all main analyses.

## Results

In total, 211 women and 59 men were included (Table 1). Among women, 52 (25%) represented higher levels of education in exercise physiology (BSc or MSc studies), and the corresponding number among men was 12 (20%).

**Table 1:** Demographic information on female and male fitness instructors included in this study.

	**Age, years**	**BMI, kg × m^**-2**^**	**PT, *n* (%)**	**GI, *n* (%)**	**Combo, *n* (%)**
Women	35.3 (11.3)	22.9 (3.3)	20 (10)	153 (73)	37 (18)
(*n* = 211)	[*18, 78*]	[*18.0, 31.6]*			
Men	39.2 (13.7)	25.9 (2.4)	16 (27)^*^	27 (46)^*^	16 (27)
(*n* = 59)	[*19, 80*]	[*20.4, 33.8*]			

*Values are mean (StD) and [range]*.

*BMI, body mass index; PT, personal trainer; GI, group instructor; Combo, operating as PT and GI*.

* *Significantly different between sexes, p ≤ 0.008*.

### Sexual Harassment

The majority of participants had never experienced any SH at the fitness centers where they were working. Still a total of 63 (30%) women and 13 (22%) men reported some sort of SH, with no between-sex differences either in this overall finding or within each SH category (*p* > 0.21) (Table 2). In the total sample, 62 (23%) reported to have experienced verbal SH, 33 (12%) had experienced nonverbal SH, and 20 (7%) had experiences of physical SH, all being statistically significant different (*p* < 0.001). Among those experiencing any SH, verbal harassment was most common (reported by 83%).

**Table 2:** Sexual harassing (SH) experiences reported by participants, separated by sex and by profession, and the reported sources.

	**Women**	**Men**	**PT**	**GI**	**Combo**
**Verbal**	52 (25%)	10 (17%)	13 (36%)	38 (21%)	11 (21%)
Customers	41 (19%)	7 (12%)	11 (31%)	30 (17%)	7 (13%)
Colleagues	20 (10%)	4 (7%)	5 (14%)	15 (8%)	4 (7.5%)
Leaders	4 (2%)	1 (2%)	2 (6%)	2 (1%)	1 (2%)
**Nonverbal**	27 (13%)	6 (10%)	4 (11%)	21 (12%)	8 (15%)
Customers	24 (11%)	4 (7%)	4 (11%)	19 (11%)	5 (9.4%)
Colleagues	7 (3%)	2 (3%)	1 (3%)	5 (3%)	3 (6%)
Leaders	1 (0.5%)	–	–	1 (1%)	–
**Physical**	16 (8%)	4 (7%)	5 (14%)	11 (6%)	4 (8%)
Customers	13 (6%)	2 (3%)	4 (11%)	9 (5%)	2 (4%)
Colleagues	3 (1%)	1 (2%)	–	2 (1%)	2 (4%)
Leaders	1 (0.5%)	1 (2%)	1 (3%)	1 (1%)	–

*Multiple choices were possible within each category (type of SH and type of perpetrator). Numbers are frequency (percent)*.

*PT, personal trainer; GI, group instructor; Combo, operating as PT and GI; Comments, sexual harassing comments (oral, written, digital); behavior, sexual harassing body language/glance/blotting or spread of private images; physical, physical touch, abuse, or rape*.

Evaluating the participants by profession, 16 (44%) of PTs, 45 (25%) of GIs, and 15 (28%) of combo had experienced some sort of SH, with no statistical difference between professions (*p* = 0.06). Customers were found to be the most frequent source for any sexual harassing behavior reported by 63 participants (23% of total sample) compared to colleagues with 27 reports (10% of total sample) and to leaders with 6 reports (2% of total sample) (Table 2). There were statistical differences in frequency of SH from clients compared to colleagues (*p* < 0.001) and from colleagues compared to leaders (*p* < 0.001), but no statistical difference between SH from clients compared to leaders (*p* = 0.5).

### Associations Between Experience of SH and Mental Health Symptoms

**Figure 2:**
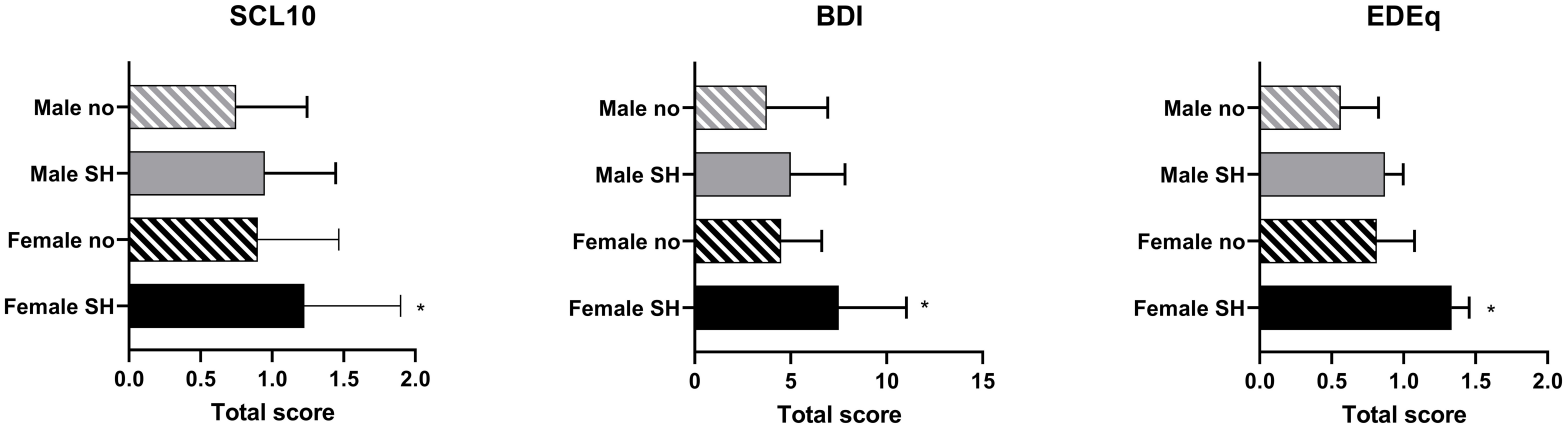
Median (IR) sex-specific scores in BDI, SCL-10, and EDE-q according to whether or not having been exposed to sexual harassment. SCL-10, symptom checklist; BDI, Beck Depression Inventory; EDEq, Eating Disorder Examination Questionnaire; Male no, males not exposed to sexual harassment; Male SH, males exposed to sexual harassment; Female no, females not exposed to sexual harassment; Female SH, females exposed to sexual harassment. *Significant difference between the two groups of females, *p* < 0.01.

Figure 2 presents scores in BDI, SCL-10, and EDE-q in women and men separately according to whether or not they have been exposed to SH. The nuanced analyses revealed higher scores in BDI, SCL-10, and EDE-q global among women having experienced verbal SH compared to women with no such experiences (*Z* = −2.4, *p* = 0.018; *Z* = −3.7, *p* < 0.001; and *Z* = −3.3, *p* = 0.001, respectively). Additionally, significantly more female GIs having experienced verbal SH scored higher in SCL-10 and EDE-q global compared to female GIs with no such experiences (*Z* = −3.4, *p* = 0.001; and *Z* = −2.5, *p* = 0.01). No other statistically significant differences according to sex or profession were found in symptoms of anxiety, depression, or eating disorders.

## Discussion

The aim of this study was to explore the frequency of work-related experiences of SH among female and male fitness instructors, to study any differences between sexes and between professions, to study the origin of SH, and to identify any differences in mental health symptoms between those with or without reported experience of SH. Corresponding to our first hypothesis, and with reference to previous findings of SH in health service professionals (i.e., frequency of 10–23%) (2, 5), we identified a comparable high frequency of SH in fitness instructors (here 22–30%). Contrasting to the second hypothesis, albeit a higher percentage of women reporting experience of SH, there was no statistical difference between women and men. According to our third and fourth hypotheses, we found customers more frequently reported as the source of perpetration, a finding further underscored by identifying PTs to more often report such experiences compared to GIs and combos. Finally, somewhat in support of our last hypothesis, in women, but not in men, there were statistically significant higher scores in symptoms of depression, anxiety, and eating disorders among those reporting experiences of SH.

Our findings render support to a previous suggestion that SH more typically occurs in relationships where the perpetrator has a higher position on the hierarchical ladder, hence implicating that workplaces provide a risk arena (12). Rather than finding the intra-organizational hierarchical structure the most typical cause of such unwanted experiences, the customers were identified as the most frequent perpetrators. Interestingly, the Norwegian Living Condition Survey, which included employees within sport, identified a lower frequency of SH among those working in service professions and in culture/sport professions (8 and 4%, respectively) compared to our results (5). An explanation to these contrasting results may be the difference in work done and roles undertaken, i.e., working at organizational levels in sport (5), compared to performing personal client/customer consultation, as in the current study. Our results compare well to the findings from Union-organized work–life surveys and national work–life surveys in Norway, exploring the frequency of SH in service professions. Here, the overall frequency of reported SH by women was 23–24% among hotel and restaurant employees and 17–23% among employees in health and welfare services, with the corresponding number of men being 10–21% (2, 5). Health professionals interact directly with clients/customers; they undertake close, physical, and often intimate interactions with the client, with a courteous and respectful approach (hence, not offending the customer/client with accuses) and a traditional viewpoint that “customers are always right.” The high-risk scenario by offering personal health services is underlined by the high frequency (47%) of SH reported by physiotherapists in a 12-month exposure survey (6). As such, findings disclose that personal service professionals are highly exposed to unwanted behavior like SH. Fitness facilities are of no exception; these represent service-oriented workplaces, in which fitness instructors offer help and support to paying customers. Within any such service-oriented workplace, customers' satisfaction is highly prioritized, which may put the employee in a challenging situation with regard to offending verbal or physical behavior and where they feel that they “just have to deal with it” (26).

Working within the fitness industry, that for decades has profited and marketed on body figure and appearance matter (14), may bring up ideas about a body appearance-fixated culture in which physical intimacy is accepted, expected, and offered. This is associated with other professions in which the body is used as a main working tool (e.g., theater, acting, sports) and may result in vague boundaries between the private body and the professional body or person (27). As much as the fitness instructors communicate their professional offers and promote themselves in social media with highly appearance-dominated imagery, clients and colleagues may believe comments on their appearance are the objective and therefore accepted or even wanted. Rather than respecting the instructors for their professional competence, the objectification of their body undertaken by themselves in their own marketing strategies, also seen in the general promotions by the fitness industry, may give an impression by the customer that verbal and nonverbal approaches toward the instructors body figure and appearance are appreciated (28). We recently reported on the high level of body figure idealization and experiences of body appearance pressure in this sample, which may underline such suggestion (15). The current results also identified verbal harassment as the most common form of SH (83% of all instructors who reported any form of SH).

Overall, our result aligns with previous observations, in which women, regardless of age, predominantly working in service and health professions, are most frequently exposed to SH and most typically report clients/customers as the perpetrator (2, 5, 6). Our finding of a rather high frequency of SH among men underlines a more sex-neutral, high-risk scenario for such experiences when offering personal health services in a body appearance-focused culture. Previous findings suggest that those who have experienced SH at the workplace often work in close relations to clients (2, 5, 6), which also may explain our nuanced finding in which PTs more frequently report experiences of SH compared to GIs and Combos. It is worth noticing that we had significantly more men compared to women that were working as PTs. Being a PT was identified with the highest frequency of SH experiences, and as such these facts may have impacted the lack of difference between sexes in reported experiences of SH in the current study. Importantly, international findings point to a higher presence of female PTs compared to male PTs (29) and may implicate a high-risk scenario (i.e., being female and a PT).

### Mental Health Symptoms

When actively taking part in the objectivization of the body, by relying on the “bodily capital,” one may question whether any sexually related behavior from others have less clinical impact compared to those *not* taking part in the objectivization of the body. Importantly, we identified a higher intensity of symptoms for depression, anxiety, and eating disorders among women with experiences of SH compared to women with no such experiences. Previous findings from women with experiences of SH at work identified a two times higher chance of reporting psychological distress 2 years later, compared to those with no such experiences (30). Also, in line with our findings, for men, such experiences did not relate to, or predict, later psychological distress. It is difficult to explain these sex-related differences in associations to exposure of SH. However, definitions of and type of SH, and the frequency and repetition or routine exposure of such, may be important explanatory variables to the sex-related inconsistent findings in mental health associations. In addition, women tend to report more mental health distress in general, specifically of symptoms directed inwardly, and to more often seek help for such, compared to men (31, 32). Still, it is also reasonable to suggest that the historical and traditional gender roles, having women subordinated and often dependent on men, and the view of women as a vulnerable offer may reinforce the personal experience as gravely degrading (31). As an extension to this, it is reasonable to speculate if the low frequency of mental health problems among men associated with challenging experiences is a result of not carrying any “burden” of a low hierarchy history, or if emotional disruption is underreported as a consequence of the male, robust ideal (2, 32). Interestingly, a previous Norwegian study found preexisting psychological distress among men to predict future SH (30). Although being speculative, this may suggest that psychologically distressed men deviate from the traditional perception of the man as a dominant, robust individual and as such makes him more vulnerable to SH. The preexistence of psychological illness is also previously suggested as a reason to why some (women) experience SH, and others not (33).

### Strengths and Limitations

The strengths of this study are using a clearly defined and nuanced (three categories) definition of SH with examples of harassing behaviors presented, including both men and women. Limiting the generalizability is the cross-sectional design, depriving us from the opportunity to suggest causes and effects of the measures. We did unfortunately not explore the knowledge by the employees on intra-organizational policy and measures toward SH. However, from observational and professional experience within the scientific group, and based on knowledge from other comparable findings, such policy and measures are in general under-communicated in the fitness industry, and few leaders in Norwegian organizations are aware of their organizational definition of SH and of established measures (34). Important to mention is the violation of the statistical assumption in our analyses between categories of profession and between categories of perpetrators (i.e., the categories being compared were not mutually exclusive).

## Conclusion

We identified an alarmingly high frequency of experiences with SH among female and male fitness instructors. Customers were identified as the most common cause to such experiences, which may complicate the process of addressing the issue. To make the professional individual confident in how to handle and act toward such unwanted experiences, the fitness industry needs to implement a common and publicly well-known policy against SH with jointly measures (4).

## Data Availability Statement

The raw data supporting the conclusions of this article will be made available at request by the authors, without undue reservation.

## Ethics Statement

The studies involving human participants were reviewed and approved by Norwegian Regional Committees for Medical and Health Research Ethics (No. 28855). The patients/participants provided their written informed consent to participate in this study.

## Author Contributions

TM initiated the research, attained ethical approvals, and administered the recruitment and communication. NS, CS-B, and JS-B contributed during the planning of the study. All authors contributed with writing the manuscript and approving final version.

## Conflict of Interest

The authors declare that the research was conducted in the absence of any commercial or financial relationships that could be construed as a potential conflict of interest.

## Publisher's Note

All claims expressed in this article are solely those of the authors and do not necessarily represent those of their affiliated organizations, or those of the publisher, the editors and the reviewers. Any product that may be evaluated in this article, or claim that may be made by its manufacturer, is not guaranteed or endorsed by the publisher.
